# The role of non-coding RNAs in regulating R-loop dynamics in cancer: Mechanisms and therapeutic implications

**DOI:** 10.1016/j.apsb.2026.06.030

**Published:** 2026-06-17

**Authors:** Sixiang Zheng, Junjie Li, Hong Lin, Xudong Zhang, Fangyi Long, Ting Wang

**Affiliations:** a Department of Clinical Research, Sichuan Clinical Research Center for Cancer, Sichuan Cancer Hospital & Institute, Sichuan Cancer Center, University of Electronic Science and Technology of China, Chengdu 610041, China; b Department of Breast Surgery, Sichuan Clinical Research Center for Cancer, Sichuan Cancer Hospital & Institute, Sichuan Cancer Center, University of Electronic Science and Technology of China, Chengdu 610041, China; c Laboratory Medicine Center, Sichuan Provincial Women's and Children's Hospital, Affiliated Women's and Children's Hospital of Chengdu Medical College, Chengdu Medical College, Chengdu 610041, China

**Keywords:** R-loop, ncRNA, Cancer, Gene regulation, Molecular mechanism, Genome instability, DNA damage response, Therapeutic resistance

## Abstract

R-loops, RNA:DNA hybrids formed during transcription, play critical roles in regulating gene expression and maintaining genome stability. Dysregulation of R-loop formation and resolution has been linked to genomic instability, a hallmark of cancer. Recent advances have highlighted the pivotal role of non-coding RNAs (ncRNAs), particularly long non-coding RNAs (lncRNAs) and circular RNAs (circRNAs), in modulating R-loop dynamics, influencing tumorigenesis, and contributing to therapeutic resistance. These ncRNAs participate in the formation, stabilization, and resolution of R-loops, which in turn regulate critical processes such as transcriptional regulation, DNA repair, and chromatin architecture. For example, circRNAs such as circSMARCA5 and circDMD have been shown to induce R-loop formation, influencing gene expression and sensitizing cancer cells to chemotherapy. Conversely, lncRNAs such as TUG1 and NEAT1 regulate R-loop resolution, maintaining genome stability and enhancing tumor cell survival. The interaction between ncRNAs and R-loops offers promising avenues for targeted therapeutic strategies aimed at restoring R-loop balance to improve cancer treatment outcomes. This review provides an in-depth exploration of the molecular mechanisms by which ncRNAs modulate R-loop dynamics and discusses their potential as biomarkers and therapeutic targets in oncology. Furthermore, we highlight the challenges and future directions in translating these findings into clinical applications.

## Graphical abstract

This review integrates how lncRNAs and circRNAs regulate R-loop dynamics in cancer, summarizing mapping strategies and therapeutic opportunities.

**Figure:**
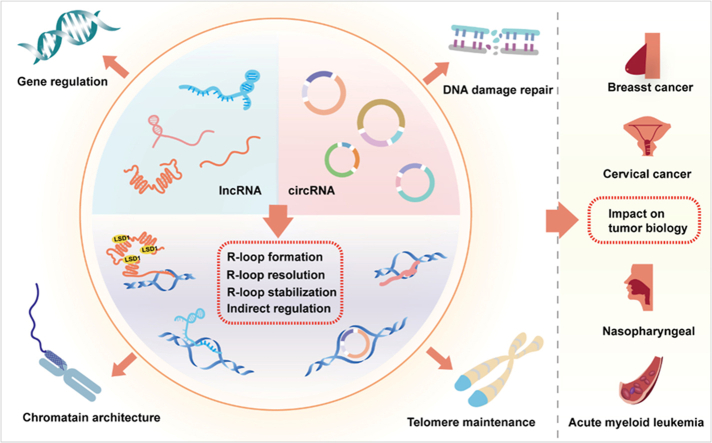

## 1 Introduction

At the forefront of oncology, studying the mechanisms of gene expression regulation is an important approach to uncovering tumorigenesis and cancer progression. Recent studies have revealed that non-coding RNAs (ncRNAs) and R-loop structures are emerging as key regulatory factors in gene expression networks. R-loops, which consist of an RNA:DNA hybrid paired with a displaced single-stranded DNA segment, are now believed to be widespread across eukaryotic genomes^1^. A growing body of evidence suggests that R-loops may function in dual capacities: under normal physiological conditions, they seem to modulate various transcriptional and epigenetic processes^2^, ^3^, ^4^. These include the regulation of transcriptional initiation, elongation, and termination, as well as influencing the chromatin state and DNA methylation patterns^5^^,^^6^. Conversely, persistent or dysregulated R-loops often result in the generation of double-strand breaks, replication stress, or chromosomal rearrangements. This instability is widely considered a key contributor to tumorigenesis^7^, ^8^, ^9^. The maintenance of R-loop homeostasis is known to be shaped by a combination of transcriptional activity, DNA sequence features, supercoiling stress, RNA processing, and helicase function. Notably, a growing number of ncRNAs have been found to regulate the dynamic balance of R-loops. They influence the formation, stability, or disassembly of R-loops by direct base pairing or by participating in the regulation of RNA-binding proteins and chromatin accessibility^10^^,^^11^. These complex regulatory mechanisms strictly control the balance of R-loops, maintain genomic stability, and prevent oncogenic transformation.

ncRNAs are generally considered to lack protein-coding ability, but they still play a vital role in gene expression regulation, chromatin remodeling, cell differentiation and disease development^12^, ^13^, ^14^. In recent years, long non-coding RNAs (lncRNAs), circular RNAs (circRNAs) and microRNAs (miRNAs) have been found to exhibit diverse and context-dependent regulatory functions in cell signaling pathways^15^, ^16^, ^17^, ^18^. Recent studies have found that ncRNAs containing small open reading frames (sORFs) can encode bioactive peptides that affect cancer cell behavior^19^. In addition, ncRNAs can regulate alternative splicing, generating multiple transcript isoforms that modulate signaling cascades involved in both the promotion and suppression of tumor progression^20^.

Multiple studies have demonstrated a close association between aberrant R-loop formation and the expression of ncRNAs, particularly circRNAs and lncRNAs^21^^,^^22^. For example, in breast cancer, overexpressed circSMARCA5 has been shown to form abnormal and stable R-loop structures with the DNA of its host gene, leading to transcriptional stalling. This disruption compromises the DNA damage repair capacity of tumor cells and consequently enhances their sensitivity to chemotherapeutic agents such as cisplatin and bleomycin^23^. Moreover, in ovarian cancer, the lncRNA PLADE, which is enriched in ascitic exosomes, interacts with HNRNPD to impair the clearance of RNA:DNA hybrids, thereby promoting R-loop accumulation and exacerbating DNA damage^24^. These findings underscore the critical roles of ncRNA-mediated R-loop dynamics in tumor cell proliferation, metastasis, and drug resistance, suggesting that this regulatory axis may serve as a promising target for cancer diagnosis and therapy.

Despite these initial advances, the precise molecular mechanisms by which ncRNAs regulate R-loops and their functional implications in cancer remain incompletely understood. In particular, how ncRNAs modulate R-loop formation, stabilization, and resolution—and how these processes influence tumor initiation, progression, and therapeutic resistance—warrants further investigation. This review summarizes the underlying mechanisms of the ncRNA–R-loop axis in cancer. Additionally, we discuss the potential of ncRNAs and R-loops as biomarkers for cancer diagnosis, disease monitoring, and prognostic evaluation, thereby offering new strategies for cancer diagnosis and targeted therapy.

## 2 Biological characteristics of ncRNAs and R-loops

### 2.1 Classification and functions of ncRNAs

The advent of high-throughput sequencing technologies and advances in bioinformatics have facilitated the identification of a vast number of ncRNAs with biological functions, and have fundamentally challenged the traditional view of genomic “non-coding” regions as transcriptional noise. Historically, ncRNAs were largely considered byproducts of transcription. However, since the discovery of the lncRNA Xist in the early 1990s—which mediates X chromosome inactivation in mammals—the essential roles of ncRNAs in gene regulation and genome stability have been increasingly recognized^25^.

**Figure 1:**
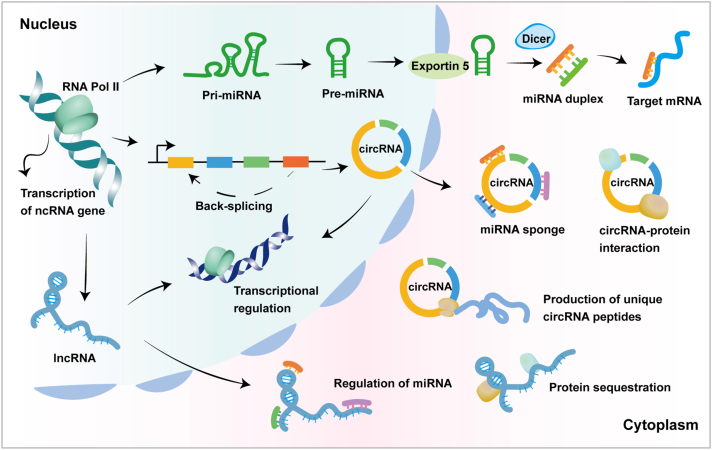
Classification and main functions of regulatory ncRNAs (miRNAs, circRNAs, and lncRNAs). The figure illustrates the biogenesis of miRNA (transcribed by Pol II, processed into pri-/pre-miRNAs, exported by Exportin 5, and cleaved by Dicer to silence mRNA), circRNA (formed by back-splicing, functioning as a miRNA sponge, interacting with proteins, and potentially encoding unique peptides), and lncRNA (transcribed in the nucleus, regulating transcription, acting as a miRNA regulator, and sequestering proteins to modulate cellular functions).

ncRNAs can be broadly classified into housekeeping and regulatory types. Housekeeping ncRNAs (*e.g.*, rRNAs, tRNAs, and snRNAs) are vital for basic cellular activities such as genetic information transmission and RNA splicing. Regulatory ncRNAs, on the other hand, constitute a complex regulatory network and include lncRNAs, circRNAs, and miRNAs (Fig. 1)^17^^,^^26^^,^^27^. The key features of housekeeping and regulatory ncRNA classes are summarized in Table 1^16^^,^^26^^,^^28^, ^29^, ^30^, ^31^, ^32^, ^33^, ^34^, ^35^, ^36^, ^37^, ^38^, ^39^, ^40^, ^41^, ^42^, ^43^. Among them, lncRNAs and circRNAs have gained particular attention because of their nuclear localization and their involvement in chromatin remodeling, transcriptional regulation, and RNA processing, all of which are closely linked to the formation and stability of R-loops^44^^,^^45^. Although miRNAs are important regulatory ncRNAs, their short length, predominant cytoplasmic localization, and limited involvement in R-loop dynamics render them less relevant to this discussion^46^. Therefore, this review focuses on lncRNAs and circRNAs, which are intimately involved in transcriptional control.

**Table 1:** Classification of housekeeping and regulatory ncRNAs and their structural and functional features.

ncRNA Class	Representative type	Key feature	Major biological function	Ref.
Housekeeping ncRNAs	rRNA	• Highly conserved • Essential for basal cellular activities • Ubiquitously expressed • Generally abundant and relatively stable	• Structural/catalytic core of ribosomes; supports protein synthesis	26,28, 29, 30
	tRNA		• Amino acid delivery and decoding of mRNA codons during translation	
	snRNA		• Pre-mRNA splicing in the spliceosome	
	snoRNA		• Guides chemical modifications (*e.g.*, 2ʹ-*O*-methylation) and maturation of other RNAs	
	7SL RNA		• Component of the Signal Recognition Particle; targets proteins to ER	
	7SK RNA		• Regulates Pol II pause release/elongation *via* P-TEFb sequestration	
	Y RNA		• Roles in DNA replication initiation and RNA quality control/stress responses	
	RNase P RNA		• Ribozyme; processes 5ʹ end of tRNA precursors	
	RNase MRP RNA		• Ribozyme; involved in pre-rRNA processing and mitochondrial DNA replication	
Regulatory ncRNAs	miRNA	• Short (∼22 nt) • Predominantly cytoplasmic • Involved in post-transcriptional regulation	• mRNA degradation and translational repression • Fine-tuning of signaling pathways	31,32
	lncRNA	• >200 nt, diverse structures • Enriched in nucleus or chromatin • Tissue- and context-specific expression	• Chromatin remodeling • Transcriptional regulation • Modulation of splicing, stability, and translation • Participation in R-loop formation and resolution	33, 34, 35, 36
	circRNA	• Covalently closed circular structure • High stability and resistance to exonucleases • Cell-type-specific	• miRNA sponges (ceRNA) • Regulation of transcription and splicing • Protein scaffolding • Some encode peptides *via* sORFs • Formation or stabilization of R-loops	16,37, 38, 39, 40
	Other regulatory ncRNAs: piRNA, eRNA, tsRNA, etc.	• Stage/tissue/stress-specific expression • Often low abundance, unstable or processing-derived • Functions are pathway- and context-dependent	• piRNA: Transposon silencing; genome stability; germline regulation • eRNA: Supports enhancer activity and enhancer–promoter communication • tsRNA: Stress-linked control of translation/RNA fate and stress-granule dynamics	41, 42, 43

lncRNAs are RNA transcripts longer than 200 nucleotides that are typically produced by RNA polymerase II (Pol II) and acquire a 5′ cap structure and a 3′ poly(A) tail after splicing^47^. Compared with mRNAs, lncRNAs are generally less abundant, have weaker sequence conservation, and exhibit higher cell- and context-specific expression, often remaining in the cell nucleus. Their complex secondary structures increase their functional diversity and stability. These characteristics enable lncRNAs to participate in diverse types of gene regulation^48^, ^49^, ^50^. Functionally, lncRNAs regulate gene expression through chromatin remodeling, transcriptional regulation, and mRNA processing^51^, ^52^, ^53^. They recruit chromatin-modifying complexes, such as histone acetyltransferases and methyltransferases, to specific genomic loci, thereby altering chromatin structure and affecting transcription^54^. In addition, lncRNAs interact with Pol II and transcription factors to modulate transcription initiation and elongation. Notably, many nuclear lncRNAs are located at promoters, transcription start sites, or replication origins, where their structured RNA domains can stabilize or disrupt RNA:DNA hybrids, thereby affecting R-loop formation, transcriptional pausing, and genome stability^55^. Furthermore, lncRNAs can also regulate alternative splicing, mRNA stability, and translation through interactions with splicing factors and RNA-binding proteins (Fig. 1)^56^, ^57^, ^58^.

circRNAs represent another major class of regulatory ncRNAs characterized by a covalently closed circular structure generated through back-splicing. In the biosynthesis of circRNAs, the downstream splice donor and upstream splice acceptor are linked by a covalent bond, a process facilitated by complementary intron sequences, RNA-binding proteins, or exon jumping events^16^^,^^37^^,^^59^. Their circular structure endows them with strong resistance to exonuclease degradation, thereby exhibiting high stability and performing a variety of biological functions. One extensively accepted mechanism is their ability to act as miRNA sponges, whereby they sequester miRNAs and relieve the repression of target mRNAs, thus increasing gene expression within the competing endogenous RNA (ceRNA) network. In addition, circRNAs can interact with DNA, Pol II and transcriptional regulators—for example, by forming RNA:DNA hybrids at regulatory sites—to modulate gene transcription and splicing. Additionally, they may serve as scaffolds for recruiting or regulating the activity of RNA-binding proteins. Emerging evidence further indicates that some circRNAs contain internal ribosome entry sites (IRESs), enabling them to encode functional peptides (Fig. 1)^16^^,^^45^^,^^60^^,^^61^.

Taken together, lncRNAs and circRNAs exert precise regulatory control over gene expression through interactions with chromatin-modifying complexes, transcriptional machinery, and RNA processing factors. These regulatory capabilities position them as key modulators of R-loop homeostasis, thereby affecting genome stability and cancer-associated signaling pathways.

### 2.2 Formation mechanism, detection techniques, influencing factors, and physiological functions of R-loops

#### 2.2.1 Formation mechanism of R-loops

**Figure 2:**
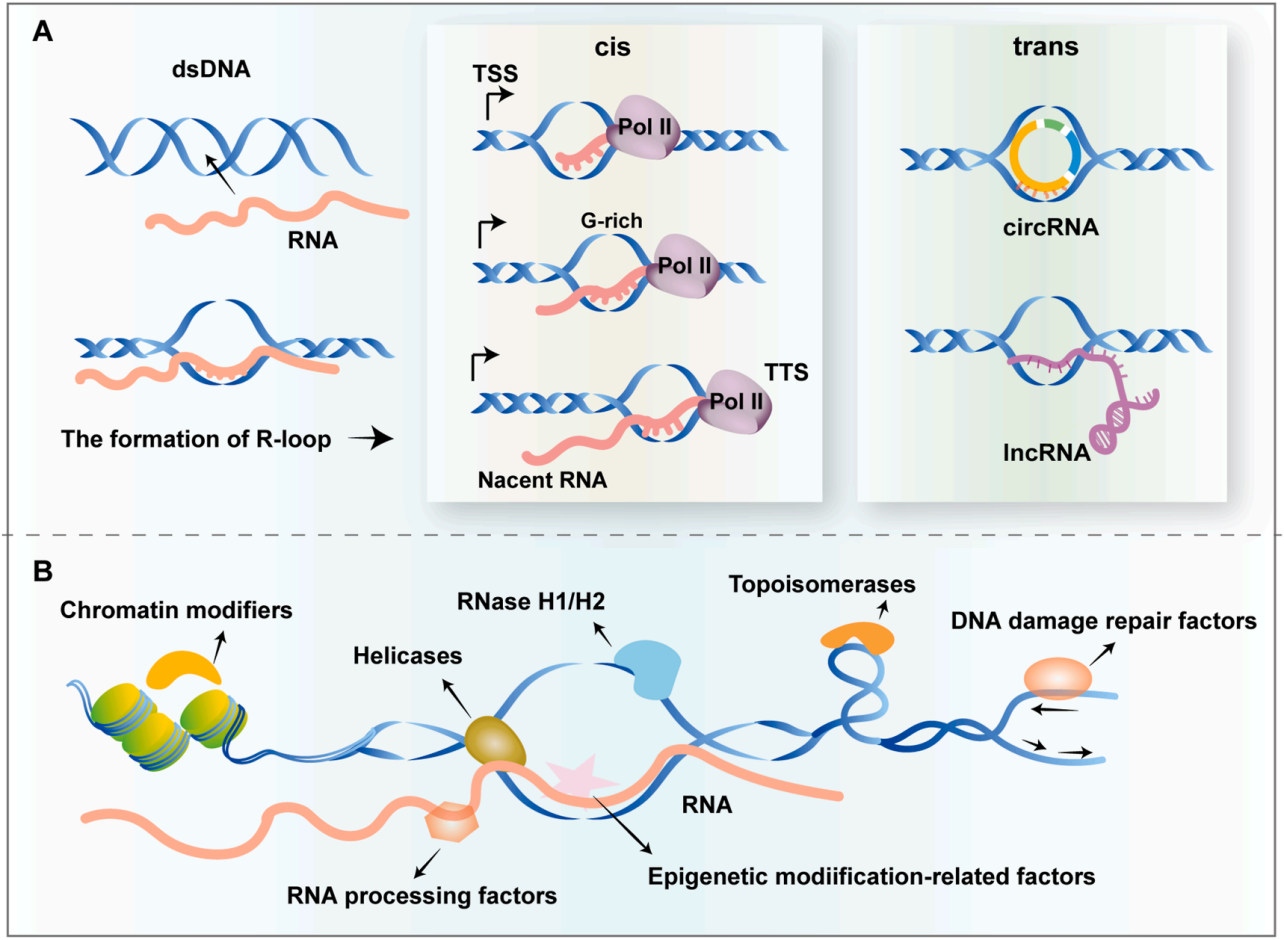
Schematic representation of R-loop formation and associated regulatory factors. (A) R-loops are three-stranded nucleic acid structures consisting of an RNA:DNA hybrid and a displaced single-stranded DNA, often formed during transcription. They are classified into two types: *cis* R-loops, which form when nascent RNA reanneals to the template DNA strand behind Pol II during transcription, and *trans* R-loops, which are typically mediated by ncRNAs. (B) Key R-loop regulatory factors include chromatin modifiers, helicases, RNase H1/H2, topoisomerases, DNA damage repair factors, RNA processing factors, and epigenetic modification-related factors. These factors regulate the formation and stability of R-loops through enzymatic actions, RNA processing, epigenetic modifications, chromatin remodeling, and DNA repair pathways.

R-loops are triple-stranded nucleic acid structures formed during transcription, consisting of an RNA:DNA hybrid and a single-stranded DNA (ssDNA) strand (Fig. 2)^62^. Genome-wide studies have shown that approximately 5% of the mammalian genome can form R-loops, with notable enrichment at transcription start sites (TSSs), transcription termination sites (TTSs), and other regions of high transcriptional activity^1^^,^^2^. Importantly, only a subset of these R-loop regions are directly associated with cancer-related genes. R-loops are commonly found in the promoters or gene bodies of classic oncogenes (such as *MYC* and *TERT*), as well as key tumor suppressor genes (including *BRCA1*, *BRCA2*, and *ATR*)^4^^,^^63^^,^^64^. Proximal promoter or intragenic R-loops at these sites are associated with oncogene activation, DNA repair pathway dysregulation, and cancer-related genomic instability^65^. However, the overall proportion of genomic sites exhibiting R-loop formation at oncogenes varies by cell type and has not yet been fully quantified.

On the basis of the origin of the RNA involved, R-loops can be classified into two types: *cis*-acting and *trans*-acting. *Cis*-R-loops arise when nascent RNA anneals to the DNA template strand behind Pol II during transcription. In contrast, *trans*-R-loops are formed when RNA transcripts from distal loci or exogenous sources hybridize with complementary DNA sequences, which is often mediated by ncRNAs (Fig. 2)^66^^,^^67^. Compared with *cis*-R-loops, *trans*-R-loops are generally more detrimental to genome stability, as they are less stringently regulated and more prone to inducing transcription‒replication conflicts and DNA damage^68^^,^^69^.

#### 2.2.2 Detection techniques

Several mainstream technologies have been developed to detect and map R-loops^70^, ^71^, ^72^, ^73^: (1) DRIP-seq (DNA–RNA immunoprecipitation sequencing): This method employs the S9.6 monoclonal antibody to specifically recognize RNA:DNA hybrids, combined with high-throughput sequencing, to identify R-loops genome-wide. (2) R-ChIP: Utilizes tagged RNase H1 protein for chromatin immunoprecipitation, enabling the identification of functional R-loop regions with improved specificity over traditional DRIP methods. (3) Single-strand DNA-enabled DRIP Sequencing (ssDRIP-seq), microfluidic affinity purification of R-loops (MapR) and other emerging methods (such as CUT&Tag-R-loop) offer higher resolution and quantitative accuracy, making them suitable for detecting dynamic changes in R-loops under drug treatment or cellular stress conditions. (4) Immunofluorescence (IF) and fluorescence *in situ* hybridization (FISH): These imaging approaches provide insights into the spatial distribution of R-loops in cells or tissues, facilitating studies on their roles in determining cell fate. Together, these evolving tools—from DRIP-seq to R-ChIP, ssDRIP-seq, and advanced imaging—are driving progress in R-loop biology. To provide an overview of the available methods, Table 2 summarizes the main R-loop detection techniques, their principles, and key features^72^^,^^74^, ^75^, ^76^, ^77^, ^78^, ^79^, ^80^.

**Table 2:** Summary of major R-loop detection techniques.

Method	Principle	Resolution	Advantage	Limitation	Typical application	Ref.
DRIP-seq	S9.6 antibody immunoprecipitates RNA:DNA hybrids, followed by sequencing	Low-medium	Widely used; robust; genome-wide coverage	Limited specificity of S9.6; low resolution; cross- reactivity with dsRNA	Verification of R-loops at candidate genes; functional studies	74,75
R-ChIP	Uses catalytically inactive RNase H1 (RNase H1-D210N) to bind R-loops *in vivo*	High	High specificity for RNA:DNA hybrids; avoids S9.6 dsRNA cross-reactivity; good resolution	Requires protein expression; may perturb R-loop homeostasis; resolution limited by sonication	High-specificity genome-wide R-loop maps; functional R-loop loci	72
ssDRIP-seq	DRIP combined with strand-specific library prep to define R-loop direction	Medium	Strand-specific; higher resolution than DRIP-seq; better localization around TSS/regulatory regions	More complex protocol; still antibody-dependent	Precise strand-resolved R-loop profiling	76
MapR	dRNase H1–MNase fusion cleaves DNA near R-loops in native chromatin	High	High resolution; low input requirement; antibody-independent	Needs fusion protein delivery; MNase bias; no strand information	Precise mapping under stress, drug treatment, or dynamic conditions	77
CUT&Tag-R-loop	R-loop antibody guides Tn5 insertion at hybrid sites	High	Very high resolution; low input; fast workflow	Depends on binder specificity; sensitive to permeabilization; new method	Low-input R-loop mapping; single-cell potential	78
Imaging-based (S9.6-IF/FISH)	Direct visualization of R-loops in cells using antibodies or hybridization probes	Cellular	Spatial localization; compatible with perturbation assays	No sequence resolution; semi-quantitative; cross-reactivity issues	Visualize R-loop localization in cells; compare R-loop levels between conditions; co-staining with DNA-damage or transcription markers	79,80

#### 2.2.3 Physiological functions

R-loops are widespread across the eukaryotic genomes and play essential regulatory roles in cellular physiology, particularly in gene expression. They perform diverse physiological functions by modulating transcription initiation, elongation, and termination. During elongation, RNA:DNA hybrids locally increase the stability and rigidity of the nucleic acid scaffold, making it more difficult for Pol II to progress and leading to transcriptional pausing and backtracking, thereby reducing downstream gene expression. In this context, factors such as Cockayne syndrome protein B (CSB) can counteract R-loop-induced Pol II stalling and help restore productive elongation^4^^,^^81^. In contrast, at TTSs, Skourti-Stathaki et al.^82^^,^^83^ reported that cotranscriptional R-loops can facilitate transcription termination, as exemplified at the *β*-globin locus, where their removal leads to transcriptional readthrough.

In addition to their direct impact on Pol II dynamics, R-loops also shape gene expression through epigenetic mechanisms and serve as a mechanistic bridge between DNA and RNA methylation. R-loop-mediated exclusion of DNA methyltransferase 3B (DNMT3B) reinforces promoter hypomethylation, whereas METTL3-dependent m^6^A deposition promotes R-loop formation at transcription termination regions, establishing a reciprocal m^6^A modification → R-loop → DNA methylation axis^7^^,^^74^^,^^84^^,^^85^. Additional effects of m^6^A on R-loop–regulated gene expression are discussed in greater mechanistic detail in Section 2.2.4.

On the other hand, dysregulated R-loops are closely associated with genomic instability and have been implicated in tumorigenesis. Excessive R-loop formation can lead to double-strand breaks (DSBs), replication fork stalling, and chromosomal rearrangements^4^^,^^62^^,^^86^. For example, Hatchi et al.^6^^,^^87^ reported that BRCA1-deficient breast cancer cells accumulate R-loops at actively transcribed loci, impairing DNA repair and increasing mutation rates. Moreover, R-loops are involved in modulating the tumor immune microenvironment. Matthew et al.^88^ reported that R-loop accumulation activates the cGAS–STING pathway, induces type I interferon expression, promotes CD8^+^ T-cell infiltration, and enhances the response to anti-PD-1 immunotherapy.

#### 2.2.4 Regulatory factors

The formation and resolution of R-loops are tightly regulated by multiple cellular mechanisms to ensure their dynamic homeostasis. Sequence composition is one of the primary determinants. DNA regions rich in guanine (G-rich), particularly those containing four or more consecutive guanine residues at the 5′ end of RNA, are thermodynamically favourable for RNA:DNA hybrid formation. Recent genome-wide mapping studies have shown that approximately 30%–60% of R-loops overlap with predicted G-quadruplex (G4) structures, particularly at GC-rich promoters, transcription start and termination sites, fragile sites, and telomeres^89^. These G4-associated R-loops are frequently enriched in the regulatory regions of highly transcriptionally active genes, including classic oncogenes such as *MYC* and *KRAS*, as well as telomere maintenance genes such as *TERT*^90^^,^^91^. Functionally, G4–R-loop coupling can stabilize transcriptional pausing, promote promoter activation, and facilitate long-range chromatin interactions^91^. However, excessive or persistent G4-associated R-loops can impair replication‒transcription coordination, leading to replication stress and genomic instability, thereby promoting the development of cancer and other diseases^92^, ^93^, ^94^. High transcription, negative supercoiling, RNA processing defects, and the presence of G4 stabilizing ligands (such as pyridostatin) further enhance G4–R-loop formation.

Multiple enzymatic and protein factors also contribute to the dynamic balance of R-loops. For example, G4–R-loop structures are dynamically dismantled by G4-resolving helicases, including BLM, WRN, DHX36, and FANCJ, which prevent their persistent stabilization^95^. In addition, the endonucleases RNase H1 and RNase H2 specifically recognize and degrade the RNA strand within R-loops, thereby dismantling the hybrid structure^96^. Helicases such as senataxin (SETX), DDX5, and DDX21 unwind RNA:DNA hybrids to facilitate R-loop resolution^97^^,^^98^. Topoisomerase I relieves transcription-induced negative supercoiling and thereby reduces R-loop formation^96^. RNA splicing factors, RNA-binding proteins (RBPs), and other RNA processing regulators can indirectly modulate R-loop dynamics by influencing RNA splicing, modification, and release from the transcriptional machinery^66^. Key DNA repair proteins such as BRCA1 and BRCA2 are capable of recognizing and removing aberrant R-loop structures, thereby preventing transcription–replication conflicts and double-strand breaks (DSBs) that would otherwise cause genomic instability^87^^,^^99^. Moreover, chromatin remodeling factors such as the Switch Defective/Sucrose Non-Fermentable (SWI/SNF) and facilitates chromatin transcription complex (FACT) complex alter nucleosome positioning and chromatin accessibility, thereby impacting transcriptional elongation and the propensity for RNA to hybridize with DNA^100^^,^^101^. Key regulatory factors and their mechanistic roles in R-loop homeostasis are summarized in Table 3^95^^,^^100^^,^^102^, ^103^, ^104^, ^105^, ^106^, ^107^, ^108^, ^109^, ^110^, ^111^, ^112^, ^113^, ^114^, ^115^, ^116^.

**Table 3:** Key regulatory factors of R-loops and their regulatory functions.

Category	Representative factor	Primary regulatory mechanism	Effect on R-loops	Ref.
RNA:DNA hybrid-specific nucleases	• RNase H1 • RNase H2	Cleave the RNA strand in RNA:DNA hybrids	Resolve transcription-associated R-loops and reduce transcription-replication (TR) conflicts/DNA damage	102,103
RNA helicases	• SETX • DDX5 • DDX21 • DHX9	Unwind RNA:DNA hybrids or associated RNA structures	Remove persistent or aberrant R-loops	104, 105, 106
G4-resolving helicases	• BLM • WRN • DHX36 • FANCJ	Unwind G-quadruplexes that stabilize R-loops	Destabilize G4-dependent R-loops and limit replication stress	95,107
m^6^A-associated regulators	• METTL3 (writer) • FTO (eraser) • YTHDF/YTHDC proteins (readers)	Install, remove or read m^6^A marks on nascent RNA	Site-specific tuning: METTL3 can promote, FTO antagonizes; readers help termination/processing	108,109
RNA-processing and transcription factors	• SRSF1 • THOC complex • spliceosome components	Coordinate co-transcriptional RNA processing and transcription dynamics	Reduce co-transcriptional R-loop formation	110,111
Chromatin remodeling complexes	• SWI/SNF • FACT	Shift nucleosome positioning and chromatin accessibility	Context-dependent: can either promote or restrict local R-loop formation/stability	100,112
DNA repair and genome stability factors	• BRCA1 • BRCA2 • ATR • FANCD2	Detect R-loop-associated DNA damage and couple removal to repair	Process R-loop-linked lesions and prevent genome instability	113, 114, 115
Topological regulatory enzymes	• Topoisomerase I/II	Relax transcription-induced DNA supercoiling	Suppress *de novo* R-loop formation	116

Epigenetic modifications constitute an important layer of R-loop regulation. *N*^6^-Methyladenosine (m^6^A)-modified nascent RNA has been implicated in modulating the hybrid-forming potential of transcripts. METTL3-mediated m^6^A deposition promotes R-loop formation at transcription termination regions by relaxing the RNA secondary structure and facilitating RNA invasion into the DNA duplex, whereas FTO-mediated demethylation counteracts this effect^101^^,^^117^. m^6^A-marked transcripts are further recognized by reader proteins including YTHDC1 and YTHDF2, which recruit transcription termination and RNA-processing factors to m^6^A-enriched loci^118^. Through this writer–eraser–reader axis, RNA methylation epigenetically tunes R-loop dynamics and indirectly modulates transcription and genome stability (Fig. 2)^117^^,^^119^^,^^120^.

These studies collectively support a sequence- and protein-centric view of R-loop regulation, suggesting that chromatin context, helicases, nucleases, and RNA modifications determine the formation and resolution of R-loops. However, emerging data indicate that specific ncRNAs can actively specify the genomic sites and fates of R-loops, revealing another RNA-centric regulatory layer and raising the questions: which ncRNAs exert this regulatory role, and through what mechanisms do they achieve this regulation?

## 3 The key role of ncRNAs in R-loop formation and regulation

ncRNAs, particularly lncRNAs and circRNAs, exemplify this RNA-centered control. Their relatively long sequences, nuclear localization, and high sequence complementarity to genomic DNA or interaction with DNA-binding protein complexes enable them to participate directly in the formation and regulation of R-loops. In contrast, other types of ncRNAs, such as miRNAs, which are generally shorter and predominantly localized in the cytoplasm, are less likely to directly engage in R-loop formation or maintenance^121^. Consequently, lncRNAs and circRNAs have emerged as central regulators in studies exploring R-loop dynamics and their connections to gene expression and genome stability. Notably, many ncRNA-mediated R-loops are formed in trans, meaning that RNA originates from a different locus than the target DNA. Although trans R-loops occur less frequently, they tend to exhibit high specificity and functional relevance, playing crucial roles in regulating gene expression, epigenetic states, and chromatin architecture^122^^,^^123^.

### 3.1 ncRNA-induced R-loop formation

Certain lncRNAs and circRNAs can directly hybridize with DNA through sequence complementarity, thus inducing R-loop formation. For example, the lncRNA TARID is complementary to the promoter region of the Tcf21 gene, enabling it to form a localized R-loop that recruits the demethylase TET1, resulting in DNA demethylation and transcriptional activation of Tcf21^124^. Owing to their covalently closed-loop structure, circRNAs exhibit resistance to exonucleases and increased nuclear stability, which may further increase their capacity to form R-loops^40^. A recent study revealed that circ-calm4 forms a circRNA-induced R-loop (circR-loop) at the *COMP* gene in pulmonary artery smooth muscle cells, thereby regulating ferroptosis pathways^125^.

Collectively, these examples suggest that lncRNAs and circRNAs act as sequence-specific “address codes” that direct R-loop formation at defined loci. However, current evidence for ncRNA-induced R-loops comes from a small number of loci and cancer models and relies heavily on overexpression or knockdown combined with S9.6- or RNase H1-based assays, making it difficult to disentangle direct R-loop induction from secondary chromatin or transcriptional changes. As a result, the overall prevalence of this mechanism and the conditions under which ncRNA-induced R-loops shift from physiological regulators of gene expression to drivers of genome instability remain important open questions.

### 3.2 ncRNA in R-loop stabilization and resolution

In addition to initiating R-loop formation, ncRNAs also participate in maintaining R-loop stability and orchestrating their timely removal, reflecting their integral roles in the full life cycle of R-loop regulation. Given that R-loops play dual biological roles—beneficial in regulating gene expression yet harmful when they accumulate aberrantly—it is essential to ensure their proper turnover and equilibrium.

ncRNAs contribute to both the stabilization and clearance of R-loops through diverse mechanisms. On the one hand, some lncRNAs stabilize R-loops by recruiting R-loop-binding proteins, forming protective complexes that shield the RNA:DNA hybrid from helicases or nucleases. For example, ANRASSF1 lncRNA forms a stable R-loop at the *RASSF1A* promoter and recruits Polycomb repressive complex 2 (PRC2), sustaining transcriptional silencing at this locus^126^. On the other hand, ncRNAs can modulate the activity or localization of R-loop-resolving enzymes such as helicases (*e.g.*, DDX5, SETX) and nucleases (*e.g.*, RNase H1), thereby facilitating the selective removal of R-loops^64^^,^^127^. Additionally, the lncRNA NORAD interacts with PUMILIO proteins to regulate R-loop accumulation in DNA repair pathways, indirectly contributing to genome stability^128^.

These findings indicate that ncRNAs can function as “architects” that nucleate or stabilize physiological R-loops and as “supervisors” that detect and resolve harmful hybrids, with the balance between these roles dictated by the cellular context. Under basal conditions, cell type–specific lncRNAs and circRNAs (such as TARID or circSMARCA5) mainly act as architects, promoting R-loops at selected regulatory elements to fine-tune transcription and chromatin structure^23^^,^^55^. Under replication stress or DNA damage, stress signaling pathways (for example ATR–CHK1 or p53) shift ncRNA programs (such as circASCC3 or NEAT1) toward supervisory functions, recruiting helicases, nucleases and RNA-binding proteins to dismantle deleterious R-loops^106^^,^^115^^,^^129^. Against this background, the next section systematically examines how such ncRNA–R-loop circuits are deployed in cancer.

## 4 Molecular mechanisms of ncRNA-mediated R-loop regulation and their roles in cancer

### 4.1 Direct induction of R-loop formation by ncRNAs to regulate the expression of tumor-associated genes

Recent studies have shown that ncRNAs, particularly circRNAs and lncRNAs, can induce or stabilize R-loop structures at specific genomic loci, thereby modulating the transcriptional activity of target genes. These processes play critical roles in tumor initiation, progression, and therapy resistance.

**Figure 3:**
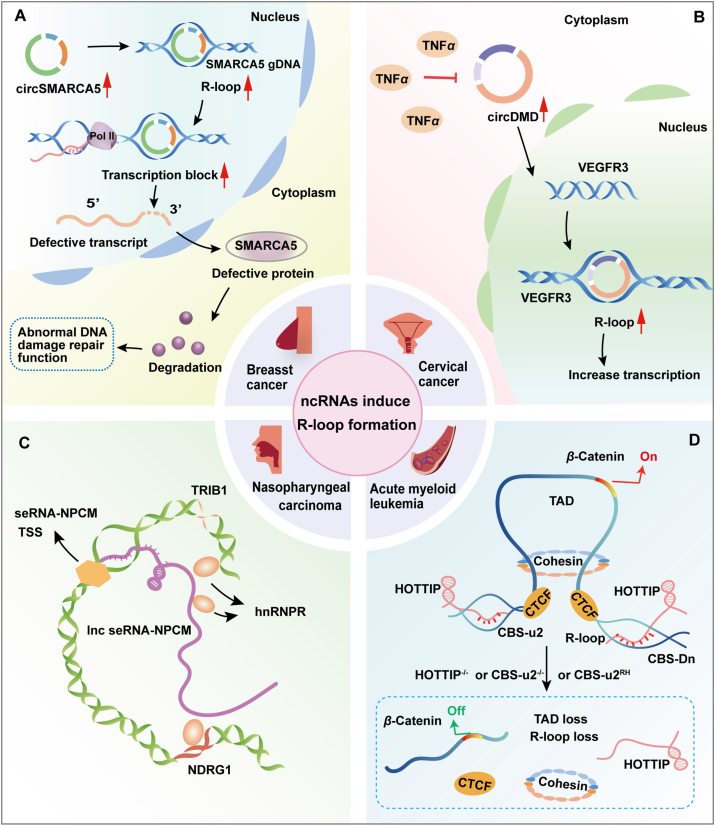
Mechanisms of ncRNA-induced R-loop formation in tumor cells. (A) circSMARCA5 in breast cancer. CircSMARCA5 induces R-loop formation at transcription sites in its parent gene, leading to transcriptional pausing and an impaired DNA damage response in breast cancer cells. (B) circDMD in cervical cancer. TNF-*α* stimulation significantly upregulates circDMD expression, which promotes R-loop formation at the *VEGFR3* promoter region and enhances its transcriptional activity. (C) lncRNA seRNA-NPCM in nasopharyngeal carcinoma. LncRNA seRNA-NPCM forms R-loop structures at the *NDRG1* promoter and interacts with hnRNPR to stabilize chromatin looping. (D) lncRNA HOTTIP in AML. The lncRNA HOTTIP facilitates R-loop formation at CTCF-binding sites (CBS-u2/Dn), reinforcing CTCF binding and maintaining the TAD structure. Additionally, HOTTIP recruits the cohesin complex and activates the oncogene *β*-catenin (*CTNNB1*).

During eukaryotic transcription, Pol II binds to gene promoters and drives transcription initiation, elongation, and termination. The formation of R-loops during the elongation phase can lead to Pol II stalling or premature termination, affecting mRNA integrity and gene expression levels. Thus, the spatial and temporal regulation of R-loops has important consequences for transcriptional output. In breast cancer, circSMARCA5 exemplifies this regulatory function by modulating transcription through R-loop formation (Fig. 3)^23^. Specifically, circSMARCA5 binds to the DNA of its host gene *SMARCA5*, inducing an R-loop structure near exon 15, which causes Pol II to pause. This process inhibits the production of full-length *SMARCA5* mRNA and promotes a truncated, nonfunctional isoform, *ΔSMARCA5*. As a member of the SWI/SNF chromatin-remodeling complex, SMARCA5 is essential for DNA damage repair. Its downregulation significantly impairs the DNA damage response in breast cancer cells. Notably, circSMARCA5 overexpression sensitizes breast cancer cells to chemotherapeutic agents such as cisplatin and bleomycin, highlighting its role as a transcriptional modulator of DNA repair capacity through R-loop formation. These findings suggest that circSMARCA5 is not only a positive regulator of R-loops but also a potential therapeutic target for overcoming chemoresistance.

In cervical cancer, circDMD is significantly upregulated upon exposure to inflammatory cytokines such as TNF-*α*. It induces R-loop formation at the promoter of the *VEGFR3* gene, thereby enhancing its transcriptional activation (Fig. 3)^130^. *VEGFR3* encodes a receptor tyrosine kinase involved in tumor angiogenesis, metastasis, and autophagy regulation. Its upregulation increases tumor cell adaptability and invasiveness^131^. Interestingly, circDMD does not act *via* the conventional miRNA sponge mechanism; rather, it directly hybridizes with DNA to form R-loops and modulates gene expression at the transcriptional level. This discovery highlights a direct structural route through which circRNAs mediate gene activation in an inflammatory microenvironment, suggesting a novel regulatory role in inflammation-driven tumorigenesis. This study identified circDMD as a structural regulatory element that links chronic inflammation and malignant transformation, providing a new perspective for targeted therapy.

Super-enhancers (SEs) are regulatory elements enriched in transcription factors and coactivators that orchestrate high expression of phenotype-determining genes through 3D chromatin reorganization^132^. In nasopharyngeal carcinoma, seRNA-NPCM, an SE-derived lncRNA, forms R-loop structures at the *NDRG1* promoter and interacts with the RNA-binding protein hnRNPR to stabilize enhancer–promoter looping (Fig. 3)^133^. NDRG1 is involved in the regulation of differentiation and metastasis, whereas hnRNPR contributes to RNA processing and chromatin modulation^134^^,^^135^. This regulatory axis also upregulates the nearby *TRIB1* gene, which participates in oncogenic signaling pathways. seRNA-NPCM promotes the expression of multiple oncogenes by structurally inducing R-loops and maintaining chromatin topology through protein interactions. Clinically, elevated seRNA-NPCM expression is correlated with poor prognosis in nasopharyngeal carcinoma, underscoring its functional importance in tumor progression and its potential as a therapeutic target or biomarker.

CCCTC-binding factor (CTCF) is a key architectural protein that defines topologically associating domain (TAD) boundaries and prevents aberrant enhancer activation. R-loops can serve as auxiliary binding platforms to enhance CTCF anchoring on chromatin. In acute myeloid leukemia (AML), the lncRNA HOTTIP induces R-loop formation at specific CTCF-binding sites to increase CTCF stability and preserve TAD integrity (Fig. 3)^136^. HOTTIP also recruits the cohesin complex—essential for chromatin looping—and cooperates with R-loop modulators to stabilize boundary regions. This facilitates long-range enhancer–promoter interactions and activates multiple oncogenes, including *β-catenin*. Functional studies have shown that disruption of CTCF-binding sites or R-loop removal compromises TAD architecture, weakens enhancer–promoter communication, and significantly inhibits leukemic cell growth and tumorigenicity. These results indicate that HOTTIP drives AML progression *via* R-loop-mediated chromatin conformation regulation and may serve as a viable therapeutic target.

Collectively, circRNAs and lncRNAs regulate gene transcription by inducing or stabilizing R-loops in specific genomic contexts, enabling precise spatial and temporal control of gene expression. However, we do not yet know whether R-loops are a central mechanism for ncRNA-driven transcriptional reprogramming in cancer or a context-dependent feature of a small subset of transcripts. In addition, current evidence comes from isolated studies in different tumor types, and there has been no systematic comparison to determine whether similar ncRNA–R-loop–transcription axes recur across cancers. Future work therefore needs to clarify how this regulatory network relates to cancer development, progression and drug resistance.

### 4.2 Regulation of R-loop resolution by ncRNAs to maintain genomic stability in cancer

Preserving R-loop equilibrium is fundamental to genomic integrity. Unresolved R-loops can lead to DNA damage and replication stress, thereby promoting tumor progression. ncRNAs can modulate the stability and distribution of R-loops by interacting with helicases (*e.g.*, DDX5, DHX9), nucleases, or other regulators involved in R-loop resolution pathways. These interactions indirectly influence the DNA damage response and the sensitivity of cancer cells to chemotherapy.

**Figure 4:**
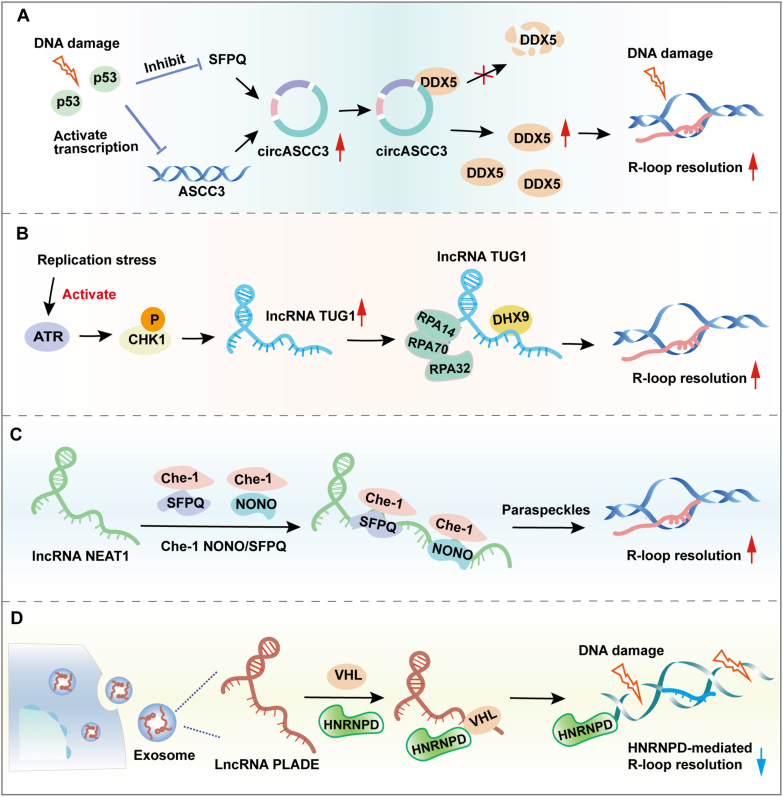
Mechanisms of ncRNA-mediated regulation of R-loop resolution in cancer cells. (A) circASCC3 in colorectal cancer. Upon DNA damage, p53 promotes the transcription of *ASCC3* while inhibiting SFPQ, leading to increased expression of circASCC3. circASCC3 binds DDX5 and protects it from proteasomal degradation, increasing DDX5 abundance and promoting DDX5-mediated R-loop resolution. (B) lncRNA TUG1 under replication stress. Replication stress activates the ATR–CHK1 signaling pathway, inducing the expression of the lncRNA TUG1. TUG1 interacts with RPA complex components (RPA14, RPA70, RPA32) and recruits DHX9 helicase to promote R-loop resolution. (C) lncRNA NEAT1 binds Che-1 and colocalizes with it paraspeckles to increase R-loop resolution. (D) lncRNA PLADE in ovarian cancer. The exosomal lncRNA PLADE binds to HNRNPD and promotes its degradation *via* a VHL-dependent ubiquitination pathway. This degradation impairs HNRNPD-mediated R-loop resolution and exacerbates DNA damage.

In colorectal cancer, p53 orchestrates circASCC3 overexpression (Fig. 4)^129^. In addition to transactivating the *ASCC3* gene, p53 also inhibits the splicing factor SFPQ, thereby specifically promoting the biosynthesis of circASCC3. CircASCC3 increases its abundance by directly binding to the RNA helicase DDX5 and protecting it from proteasomal degradation. DDX5 is a core R-loop resolvase that recognizes and unwinds RNA:DNA hybrids to maintain genomic homeostasis^137^. By stabilizing DDX5, circASCC3 enhances R-loop clearance, thereby alleviating DNA damage and genomic instability. The circASCC3–DDX5 axis not only enhances DNA damage tolerance but also exerts chemoprotective effects in tumor cells. Clinically, elevated circASCC3 is associated with poor prognosis in patients with colorectal cancer, making it a promising target for reversing R-loop-mediated chemoresistance.

Replication stress rapidly induces lncRNA TUG1, which accumulates at nuclear R-loop foci (Fig. 4)^138^. Mechanistically, TUG1 scaffolds resolution complexes: its interaction with phosphorylated RPA32 identifies vulnerable genomic sites whereas 3ʹ domain-mediated DHX9 binding recruits and activates this essential helicase. DHX9 functionality critically depends on ncRNA partnerships such as TUG1^139^. Loss of TUG1 results in abnormal R-loop accumulation, increased *γ*-H2AX levels, DNA double-strand breaks, and S/G2-phase cell cycle arrest, ultimately leading to suppressed proliferation and apoptosis in cancer cells. These findings highlight the critical role of TUG1 in maintaining R-loop homeostasis and DNA replication integrity and underscore its potential as a therapeutic target in tumors with elevated R-loop burden.

Multiple myeloma studies have revealed that NEAT1 governs R-loop resolution *via* the transcriptional cofactor AATF/Che-1 (Fig. 4)^140^. Che-1 directly binds NEAT1 and colocalizes with it within nuclear paraspeckles, thereby anchoring the complex to specific genomic loci and facilitating the timely resolution of R-loops. Loss of Che-1 results in excessive R-loop accumulation and robust activation of the interferon signaling cascade, leading to heightened systemic inflammation and correlating with poor clinical outcomes in multiple myeloma patients. Moreover, the elevated unfolded protein response commonly observed in myeloma cells further drives R-loop formation and interferon pathway activation, underscoring the bidirectional interplay among ncRNAs, cellular stress responses, and immune signaling in the pathological regulation of R-loops.

Another study revealed that the lncRNA PLADE, which is derived from the ascites-derived exosomes of ovarian cancer patients, plays an important role in regulating R-loop clearance and chemotherapy sensitivity (Fig. 4)^24^. PLADE binds to the RNA-binding protein HNRNPD and promotes its VHL-dependent ubiquitination and proteasomal degradation, thereby attenuating HNRNPD's R-loop resolution function. This leads to R-loop accumulation and enhanced DNA damage signaling, significantly sensitizing ovarian cancer cells to platinum-based drugs such as cisplatin. Notably, PLADE can be transferred intercellularly *via* exosomes, transmitting chemosensitizing effects. Both *in vitro* and *in vivo* models have shown that high PLADE expression is correlated with improved chemotherapy response, indicating its potential as a therapeutic enhancer of treatment sensitivity.

Collectively, ncRNAs modulate the expression, stability, or subcellular localization of proteins involved in R-loop clearance, thereby participating in the regulation of genomic integrity and cell fate. These mechanisms not only help explain variations in tumor responses to chemotherapeutic agents but also provide new strategies to restore DNA repair pathways and enhance therapeutic sensitivity through the regulation of R-loop balance. Targeting these ncRNA–R-loop–protein regulatory axes may offer novel breakthroughs in precision cancer therapy.

### 4.3 Roles of m^6^A-modified ncRNAs in R-loop regulation in cancer

m^6^A, a dominant post-transcriptional modification in eukaryotic RNAs, critically influences RNA stability, splicing, localization, and translation^13^^,^^141^. Emerging research now positions m^6^A as a key regulator of R-loop formation and homeostasis, adding a significant layer to the ncRNA–R-loop regulatory axis.

**Figure 5:**
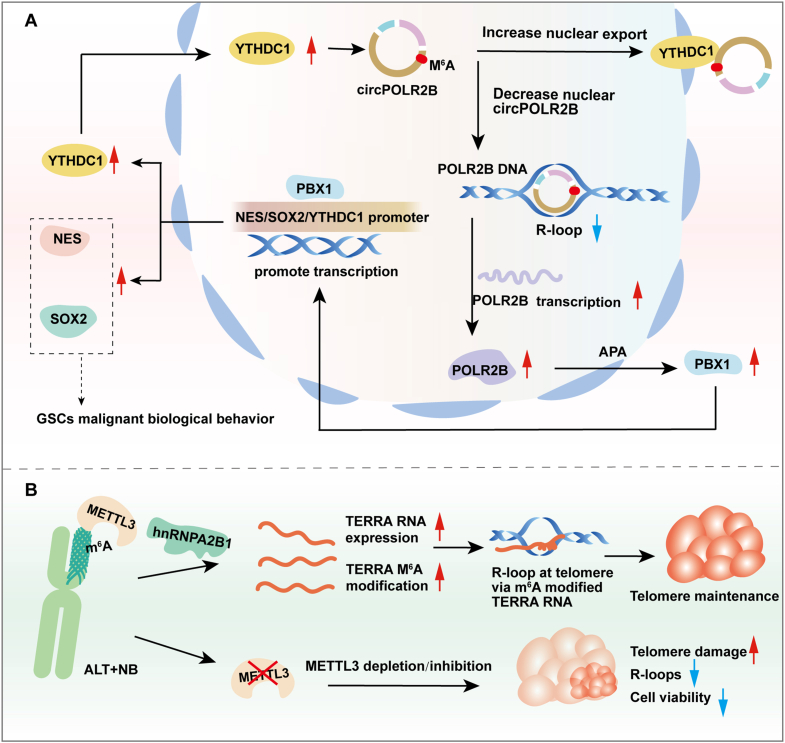
Mechanisms by which m^6^A-modified ncRNAs regulate R-loops in tumor cells. (A) circPOLR2B in glioma. YTHDC1 promotes the nuclear export of m^6^A-modified circPOLR2B, thereby limiting R-loop accumulation. Increased POLR2B transcription upregulates PBX1, which in turn activates stemness-related markers (*e.g.*, SOX2 and NES), forming a circPOLR2B–POLR2B–PBX1 positive feedback loop that drives glioma stem cell properties. (B) TERRA in ALT-positive neuroblastoma patients. METTL3-mediated m^6^A modification of TERRA enhances its interaction with hnRNPA2B1, facilitating R-loop formation at telomeres. Inhibition of METTL3 reduces telomeric R-loops, increases telomeric DNA damage, and diminishes tumor cell viability.

In gliomas, the m^6^A-modified circRNA circPOLR2B forms R-loops with its host gene *POLR2B*, directly influencing glioma stem cell (GSC) behavior (Fig. 5)^142^. Crucially, the m^6^A reader YTHDC1 facilitates the export of circPOLR2B from the nucleus. This export reduces nuclear circPOLR2B levels, diminishing R-loop formation at the *POLR2B* locus and consequently releasing the transcriptional repression of POLR2B. Downstream, POLR2B activates the transcription factor PBX1 through alternative polyadenylation (APA), which in turn induces the expression of the stemness markers NES and SOX2, forming a circPOLR2B–POLR2B–PBX1 positive feedback loop. This circuit significantly enhances GSC self-renewal, migration, and resistance to apoptosis. These findings highlight a pivotal role for m^6^A in regulating R-loop-dependent cancer stemness.

With respect to telomere regulation, telomeric repeat-containing RNA (TERRA) is a lncRNA transcribed from telomeric regions that forms R-loops with telomeric DNA and is essential for telomere maintenance, especially in telomerase-negative cells *via* homologous recombination-mediated telomere elongation^143^. The methyltransferase METTL3 catalyzes m^6^A modifications in the subtelomeric region of TERRA, which are recognized by YTHDC1 to increase TERRA stability and maintain its R-loop-forming capacity^144^. Knockdown of METTL3 or YTHDC1 accelerates TERRA degradation, significantly reduces R-loop levels, and impairs homologous recombination-mediated telomere elongation, leading to telomere shortening and dysfunction.

This m^6^A-dependent TERRA regulation is especially significant in alternative lengthening of telomeres (ALT)-positive neuroblastoma. Here, METTL3 deposits m^6^A marks on both the UUAGGG repeats and subtelomeric regions of TERRA. These modifications promote the binding of TERRA to hnRNPA2B1, enabling its precise recruitment to telomeres (Fig. 5)^143^. This interaction drives precise TERRA recruitment to telomeres, enhances R-loop formation, and activates RAD51-mediated homologous recombination. Functional assays demonstrated that METTL3 depletion results in R-loop loss, increased telomere damage, and ALT pathway suppression. Notably, ALT-positive neuroblastoma cells exhibit high sensitivity to METTL3 inhibitors, suggesting that this mechanism not only regulates telomere stability but also holds potential for targeted therapy.

Taken together, m^6^A modifications modulate the nuclear stability and localization of ncRNAs and thereby shape their capacity to form R-loops. Yet current evidence has only begun to link m^6^A-marked ncRNAs to R-loop behavior, and key mechanistic questions remain. Most studies perturb m^6^A writers, erasers, or readers and then monitor ncRNA abundance, localization, and R-loops in parallel, making it difficult to disentangle direct effects of the m^6^A mark on individual lncRNAs or circRNAs from indirect consequences of their effector proteins. It therefore remains unclear whether m^6^A influences R-loops mainly by altering ncRNA structure, stability or nuclear retention, or instead by recruiting or excluding m^6^A readers that interact with helicases, nucleases, or Pol II.

### 4.4 Stabilization of R-loops by ncRNAs to preserve chromatin architecture and telomere integrity in cancer

**Figure 6:**
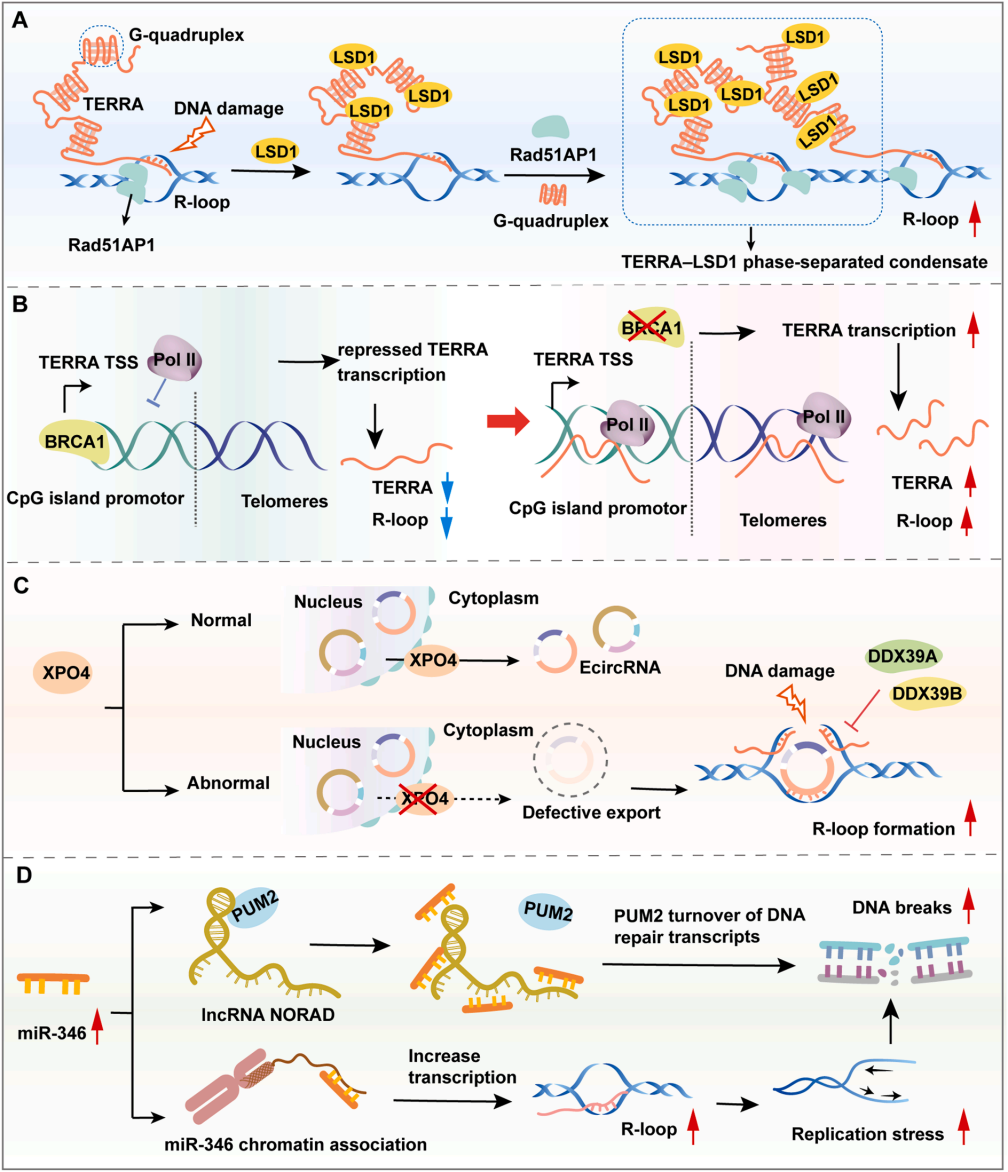
Additional ncRNA-mediated mechanisms regulating R-loops in tumor cells. (A) TERRA interacts with LSD1 to form phase-separated TERRA–LSD1 condensates, which recruit Rad51AP1 and stabilize R-loops at specific genomic loci. (B) BRCA1 binds to CpG-rich promoter regions of TERRA, repressing its transcription, reducing TERRA levels, and thereby decreasing R-loop accumulation. BRCA1 deficiency leads to TERRA overexpression and excessive R-loop formation. (C) Under normal conditions, XPO4 mediates the nuclear export of EcircRNAs. Under abnormal conditions such as XPO4 loss, EcircRNAs are retained in the nucleus, where they promote R-loop formation and DNA damage. The helicases DDX39 A/B can resolve these hybrids to mitigate DNA damage. (D) miR-346 disrupts the NORAD–PUM2 interaction, impairing DNA damage repair. Additionally, miR-346 directly binds chromatin to initiate transcription and promote R-loop formation, leading to replication stress and increased DNA double-strand breaks.

In cancer cells relying on the ALT pathway, the telomeric repeat-containing RNA TERRA stabilizes R-loop structures and promotes their enrichment at telomeric regions through interaction with lysine-specific demethylase 1A (LSD1) (Fig. 6)^145^. LSD1 binds to the G4 structure of TERRA *via* its RNA-binding domain, facilitating TERRA's localization at telomeres and promoting the formation of TERRA–LSD1 phase-separated condensates. These condensates serve as scaffolds that enrich R-loop regulatory proteins such as Rad51AP1, further enhancing R-loop formation at telomeres. Notably, LSD1 operates here independently of its classical demethylase function, acting instead as an RNA-binding structural organizer. In support of this paradigm, experimental tethering of LSD1 to telomeres rescues ALT deficiencies caused by LSD1 loss—including impaired telomere clustering and DNA synthesis defects—validating the functional importance of this complex. This LSD1-mediated organization presents a promising therapeutic vulnerability for ALT-positive tumors.

In addition, TERRA can form specific RNA:DNA hybrids with short telomeres through its UUAGGG repeat sequence, a process dependent on the recombinase RAD51 and its cofactor BRCA2^146^. RAD51 not only binds directly to TERRA but also facilitates R-loop formation *in vitro*, enabling TERRA to be selectively recruited to particular telomeres. In parallel, proteins such as RNase H1 and TRF1 act to prevent excessive R-loop accumulation, maintaining a dynamic balance crucial for telomere function.

A further study revealed that the cooperative interaction between TERRA and RAD51AP1 promotes the coexistence of R-loops and G4 structures at telomeric regions, thereby activating the break-induced replication (BIR) pathway, which is essential for sustaining ALT activity^147^. This composite mechanism shows enhanced adaptability in telomere maintenance, particularly in the absence of RAD52, suggesting that TERRA is capable of modulating telomeric repair strategies in response to specific genetic backgrounds.

Overall, TERRA not only serves as a ncRNA template for R-loop formation but also collaborates with various proteins to construct higher-order nucleic acid architectures that sustain telomere integrity and support the proliferative capacity of cancer cells. Investigating such ncRNA–protein–nucleic acid structural complexes provide a paradigm for understanding the spatial regulation of R-loops and opens new avenues for precision therapies targeting ALT-positive malignancies.

### 4.5 Indirect regulation of R-loops by ncRNAs under disrupted cellular homeostasis in cancer

The formation and clearance of R-loops are highly sensitive to various endogenous stress conditions, including imbalances in nucleic acid metabolism, inflammatory signaling, impaired RNA export, and aberrant non-coding transcription^4^^,^^69^^,^^148^. In these contexts, certain ncRNAs do not directly participate in the structural formation of R-loops but instead modulate the microenvironment that governs R-loop dynamics. This leads to abnormal R-loop accumulation, which can disrupt genome stability, activate the DNA damage response, or trigger systemic inflammation^149^. Such indirect regulatory mechanisms have been observed in various cancers and neurological disorders, highlighting the complex interplay between ncRNA regulatory networks and R-loop biology.

In the context of telomere maintenance, BRCA1 has been shown to regulate the removal of R-loops at telomeric regions *via* interactions with TERRA, a telomeric repeat-containing RNA (Fig. 6)^64^. BRCA1 binds TERRA through its N-terminal nuclear localization sequence, preventing excessive R-loop formation and telomeric DNA damage. This function involves interactions with the shelterin complex and the binding of BRCA1 to CpG-rich promoter regions of TERRA, leading to transcriptional repression and RNA degradation. This process is likely coordinated with RNA clearance factors such as SETX and XRN2. BRCA1 deficiency or mutation results in TERRA overexpression, R-loop accumulation, and telomeric replication stress, further exacerbating genomic instability. These findings underscore the multifaceted role of BRCA1 in maintaining R-loop equilibrium through ncRNA regulation.

In the case of exonic circular RNAs (ecircRNAs), Exportin 4 (XPO4) functions as a nuclear export factor that binds ecircRNAs and facilitates their translocation to the cytoplasm (Fig. 6)^150^. Loss of XPO4 results in the nuclear accumulation of ecircRNAs, which in turn form circRNA:DNA hybrids (ciR-loops). These ciR-loops further promote linear RNA:DNA hybrid formation, collectively inducing DNA damage and genomic instability. The helicases DDX39 A/B are capable of resolving these hybrids, thereby mitigating damage. However, downregulation of splicing factors such as SF3A1 or SRSF1 enhances ecircRNA biogenesis, exacerbating ciR-loop accumulation. In mouse models, XPO4 haploinsufficiency leads to male infertility and neuronal loss, underscoring the critical role of this pathway in reproductive and neural homeostasis. This mechanism offers new insights into the pathological origins of R-loop accumulation from the perspective of RNA nuclear export.

In prostate cancer, miR-346 promotes R-loop accumulation through multiple mechanisms (Fig. 6)^151^. It disrupts the interaction between the lncRNA NORAD and PUM2, thereby releasing PUM2 to degrade DNA repair–associated mRNAs and compromising the ability of the cell to resolve DNA damage. Additionally, miR-346 directly binds chromatin to initiate transcription and facilitate R-loop formation. These effects increase replication stress and double-strand breaks, ultimately activating the DNA damage response pathway. In contrast, NORAD promotes miR-346 degradation *via* target-directed miRNA degradation, maintaining genomic integrity. Notably, elevated miR-346 expression is associated with a favourable prognosis in prostate cancer patients and enhances tumor sensitivity to PARP inhibitors, suggesting that miR-346 is a promising target for precision therapy.

In another mechanism, SPT6 deficiency causes aberrant distribution of H3K36me3 histone marks and induces widespread aberrant transcription of lncRNAs^152^. These unprocessed lncRNAs bind chromatin and promote R-loop formation. When these R-loops overlap with DNA replication origins, they markedly intensify replication–transcription conflicts, leading to heightened genomic instability.

Collectively, these findings reveal that ncRNAs can indirectly regulate R-loops by altering the cellular homeostatic landscape rather than by acting solely as structural components. This indirect regulation contributes to the activation of inflammatory responses, DNA damage signaling, and transcriptional stress pathways. These insights expand the functional map of R-loop regulation and offer novel therapeutic entry points for diseases such as cancer and neurodegeneration.

## 5 Potential of ncRNAs and R-loops in tumor diagnosis and therapy

The formation and resolution of R-loops play a central role in maintaining genome stability and regulating the balance between DNA replication and transcription. Dysregulation of these processes has been widely implicated in tumorigenesis and cancer progression. Clinical studies have demonstrated that R-loop scores are closely correlated with the molecular features of various malignancies, including *KRAS* and *TP53* mutations, tumor stage, metastatic potential, and patient survival. Notably, within the tumor immune microenvironment, patients with low R-loop scores often display a T-cell exhaustion phenotype, indicative of pronounced immunosuppression^153^. Furthermore, R-loop accumulation can induce cytoplasmic RNA:DNA hybrid aggregates that activate innate immune pathways such as the cGAS–STING pathway, thereby mediating inflammatory responses and programmed cell death, which further influences tumor immune evasion and therapeutic outcomes^6^^,^^153^. Abnormal R-loop dynamics also play a critical role in drug resistance mechanisms. For example, upregulation of poly(ADP-ribose) polymerase 1 (PARP1) promotes R-loop clearance, contributing to resistance against topoisomerase I inhibitors^154^. Conversely, aberrant expression of factors such as SYCP2 enhances R-loop-mediated DNA double-strand break repair pathways, which are closely associated with resistance to antibody‒drug conjugates and platinum-based chemotherapy in breast and ovarian cancers^155^.

ncRNAs, as key components of the R-loop regulatory network, have recently emerged as promising therapeutic targets in oncology^156^. Various lncRNAs and circRNAs finely modulate R-loop formation and stability through multiple mechanisms, including the regulation of Pol II elongation, recruitment of RNA-binding proteins (*e.g.*, FUS and DDX5), and control of RNA splicing and transcription termination. These ncRNA–R-loop axes not only regulate tumor cell proliferation but are also closely associated with transcriptional adaptability, genomic instability, and epigenetic reprogramming. Targeting these pathways represents a potential strategy to overcome current therapeutic bottlenecks, such as chemoresistance, radioresistance, and resistance to targeted agents, particularly in solid tumors, including breast, colorectal, and liver cancers.

At the clinical application level, ncRNA-targeting strategies are progressively transitioning from mechanistic validation to translational implementation. Multiple studies have confirmed that certain circRNAs are stably expressed in biofluids, such as plasma and saliva, and exhibit high diagnostic sensitivity and specificity. For example, circRNA_0000392 has been proposed as a biomarker for early screening and recurrence risk assessment in patients with colorectal cancer^157^. Therapeutically, the development of ncRNA-targeting agents *via* high-throughput screening, small-molecule arrays, and structure-guided design is actively advancing^158^. Some compounds have been shown to selectively modulate ncRNA expression; for example, low concentrations of bisphenol A (BPA) and diethylstilbestrol (DES) significantly induce lncRNA HOTAIR expression in breast cancer cells *in vitro*^159^. Antisense oligonucleotides and siRNAs targeting oncogenic lncRNAs such as HOTAIR and MALAT1 have entered preclinical stages, demonstrating promising tumor suppressive effects in animal models^160^.

Breakthroughs have also been made in circRNA-based therapeutic strategies. Notably, RXRG001 is the world's first circRNA drug to enter clinical trials aimed at modulating tumor immune functions and treating radiation-induced salivary gland hypofunction^161^. Another circRNA candidate, HM2002, is undergoing clinical evaluation for ischemic heart failure and holds potential as a novel therapy for ischemic heart disease^162^. These advances not only validate the multifunctionality of circRNAs in protein-coding, immune regulation, and targeted therapy but also provide a paradigm for future ncRNA drug development.

The integration of single-cell multiomics, clustered regularly interspaced short palindromic repeats (CRISPR)-based functional screening, and AI-assisted drug design is expected to comprehensively delineate the spatiotemporal interactome of ncRNAs and R-loops. Such advances will enable precise, multilayered interventions and personalized treatment strategies, thereby driving the evolution of cancer diagnosis and therapy towards increased dimensionality and efficacy.

## 6 Conclusions and future perspectives

This review systematically summarizes and critically discusses the diverse mechanisms by which ncRNAs regulate the formation, stabilization, and resolution of R-loops, highlighting their pivotal roles in tumor initiation, progression, and therapeutic responses. Accumulating evidence indicates that lncRNAs and circRNAs can act as structural scaffolds, regulatory molecules, or platforms for protein recruitment, participating in a broad range of biological processes, such as transcriptional regulation, telomere maintenance, DNA repair, and epigenetic modulation. These findings underscore the central role of the ncRNA–R-loop regulatory network in maintaining genome homeostasis.

The functional heterogeneity of ncRNA–R-loop axes across different cancer types further provides a molecular basis for tumor classification and personalized therapeutic strategies. For example, circSMARCA5 exploits R-loop formation to silence its own host gene *SMARCA5*. In contrast, circDMD and seRNA-NPCM hijack R-loops to activate oncogenes at promoters. HOTTIP uses chromatin-associated R-loops to fortify the boundaries of topologically associating domains (TADs), maintaining genome architecture^23^^,^^130^^,^^136^. These mechanisms have been experimentally validated in breast cancer, cervical cancer, nasopharyngeal carcinoma, and AML, reflecting the high plasticity and disease specificity of R-loop-mediated transcriptional regulation in diverse tissue contexts. On the basis of the above research advances, this review further summarizes the reported representative lncRNAs and circRNAs and their mechanisms of R-loop regulation (Table 4^23^^,^^24^^,^^64^^,^^129^^,^^130^^,^^133^^,^^136^^,^^138^^,^^140^^,^^142^^,^^144^^,^^145^^,^^150^^,^^151^).

**Table 4:** Representative ncRNAs that regulate R-loop dynamics in cancer.

Name	Regulatory mode	Target/mechanism	Biological function	Cancer type	Ref.
circSMARCA5	Induces R-loop formation	Forms R-loop at *SMARCA5* gene, stalls Pol II, reduces full-length *SMARCA5* mRNA	Impairs DNA repair, increases sensitivity to cisplatin/bleomycin	Breast cancer	23
circDMD	Induces R-loop formation	Forms R-loop at *VEGFR3* promoter, activates transcription	Promotes angiogenesis, metastasis	Cervical cancer	130
circASCC3	Promotes R-loop resolution	Stabilizes DDX5 helicase, enhances RNA:DNA hybrid unwinding	Reduces R-loop accumulation, maintains genome stability	Colorectal cancer	129
circPOLR2B	m^6^A-dependent R-loop regulation	Forms R-loop with *POLR2B*, regulates PBX1/SOX2 stemness pathway	Promotes stemness, invasion	Glioma	142
ecircRNAs (XPO4-dependent export)	Prevents nuclear ciR-loop accumulation	XPO4 exports ecircRNAs, preventing nuclear R-loop formation	Maintains genome stability	Multiple cancers, infertility models	150
seRNA-NPCM (lncRNA)	Induces R-loop formation and stabilizes chromatin looping	Forms R-loop at NDRG1 promoter, recruits hnRNPR to maintain enhancer–promoter interaction	Activates oncogenes, promotes metastasis	Nasopharyngeal carcinoma	133
HOTTIP (lncRNA)	Induces chromatin-associated R-loop formation	Forms R-loop at CTCF-binding sites, maintains TAD boundaries, recruits cohesin	Activates *β*-catenin and other oncogenes	Acute myeloid leukemia	136
TUG1 (lncRNA)	Promotes R-loop resolution	Recruits DHX9 helicase *via* interaction with RPA complex	Prevents replication stress, maintains genome integrity	Multiple cancers	138
NEAT1 (lncRNA)	Promotes R-loop resolution	Interacts with Che-1 in paraspeckles to resolve R-loops	Maintains genome stability, regulates immune response	Multiple myeloma	140
PLADE (lncRNA)	Inhibits R-loop resolution	Promotes degradation of HNRNPD, impairs R-loop removal	Increases DNA damage, enhances platinum sensitivity	Ovarian cancer	24
TERRA (lncRNA)	m^6^A-dependent R-loop regulation	METTL3/YTHDC1 enhance TERRA stability and telomeric R-loop formation	Maintains telomere length and ALT activity	ALT-positive neuroblastoma, other ALT cancers	144
TERRA (lncRNA)	Stabilizes telomeric R-loops	Interacts with LSD1, RAD51AP1 to form phase-separated condensates	Sustains telomere integrity	ALT cancers	145
TERRA (*via* BRCA1)	Prevents excessive telomeric R-loops	BRCA1 represses TERRA transcription, limits R-loop accumulation	Reduces telomeric replication stress	BRCA1-deficient cancers	64
NORAD (lncRNA)	Indirect regulation of R-loops	Sequesters PUM2 to stabilize DNA repair mRNAs	Maintains genome integrity	Prostate cancer	151
miR-346	Indirect regulation of R-loops	Disrupts NORAD–PUM2 interaction, reduces DNA repair	Increases DNA damage, enhances PARP inhibitor sensitivity	Prostate cancer	151

Notably, research on ncRNA–R-loop interactions has expanded beyond oncology into various non-cancerous diseases, including pulmonary hypertension, impaired wound healing in diabetes, and neurodegenerative disorders. For example, circLrch3 promotes inflammasome activation *via* R-loop formation in pulmonary arterial smooth muscle cells, whereas Lnc530 modulates R-loop homeostasis to safeguard genomic integrity in embryonic stem cells^163^. These findings highlight the broad adaptability and therapeutic potential of ncRNA–R-loop networks in multiple pathological systems.

Despite rapid advances, several key scientific challenges remain. First, R-loop formation is highly spatiotemporally specific and dynamically regulated by chromatin architecture, transcriptional activity, and RNA secondary structure. Current detection techniques still face limitations in terms of molecular resolution, sensitivity to dynamic changes, and applicability to low-input clinical samples. Second, the inherent transcriptional complexity and alternative splicing of ncRNAs—particularly within the tumor microenvironment—complicate the elucidation of causal relationships between specific RNA structural domains and their R-loop-forming capacities. Moreover, the lack of clear biomarkers to distinguish “functional” R-loops from “pathological” accumulations hampers the identification of disease-relevant mechanisms. Some pathogenic R-loops may exert harmful effects only in the presence of specific RNA-binding proteins or modifying enzymes, presenting significant hurdles for mechanistic dissection and therapeutic target validation.

From a translational perspective, integrating large-scale multiomics data from clinical cohorts with long-term follow-up studies may facilitate the development of ncRNA–R-loop signatures as novel biomarkers for early cancer diagnosis, prognosis, and therapeutic response prediction. A precise understanding of the mechanisms governing R-loop formation and maintenance also opens avenues for the design of targeted therapeutic strategies. These may include small molecules that selectively dismantle pathogenic R-loop structures or gene editing tools to modulate the expression of critical ncRNAs, thereby reshaping the transcriptional landscape of tumor cells. Furthermore, combining R-loop-targeted therapies with conventional chemotherapy, radiotherapy, or immunotherapy may offer synergistic effects and help overcome the limitations of monotherapies.

The ncRNA–R-loop axis holds promise as a central node in the next generation of targeted intervention strategies. On the one hand, small-molecule compounds and antisense oligonucleotides could be leveraged to modulate the formation or resolution of structural R-loops with high specificity. On the other hand, coupling ncRNA-editing platforms with regulators of R-loop dynamics—such as helicases and RNA-binding proteins—could enable controllable modulation of cancer phenotypes. Moreover, integrating R-loop stability metrics, ncRNA expression profiles, and patient clinical parameters may facilitate the construction of precision diagnostic and predictive models, accelerating the translation of basic discoveries into clinical applications.

In summary, ncRNAs orchestrate R-loop biology through multiple mechanisms, critically influencing gene expression, genome stability, and cell fate. As our understanding deepens and detection technologies evolve, targeting the ncRNA–R-loop axis presents exciting, tangible opportunities—not only for novel cancer therapies, but also for truly personalized approaches. The critical next step? Deciphering the precise regulatory code and functional context-dependency of this network. Cracking this code is what will ultimately bridge the gap between fundamental discovery and impactful clinical translation.

## Author contributions

Sixiang Zheng: Writing – original draft, Visualization, Investigation. Junjie Li: Writing – review & editing. Hong Lin: Writing – review & editing. Ting Wang: Conceptualization, Supervision, Writing – review & editing. Fangyi Long: Conceptualization, Supervision. Xudong Zhang: Resources, Data curation.

## Conflicts of interest

The authors declare no conflicts of interest.
